# Budget impact analysis of increasing prescription of renin-angiotensin system inhibitors drugs to standard anti-hypertensive treatments in patients with diabetes and hypertension in a hypothetical cohort of Malaysian population

**DOI:** 10.1371/journal.pone.0212832

**Published:** 2019-02-28

**Authors:** Nurul-Ain Mohd-Tahir, Shu-Chuen Li

**Affiliations:** 1 School of Biomedical Sciences and Pharmacy, Faculty of Health, University of Newcastle, Callaghan, New South Wales, Australia; 2 Faculty of Pharmacy, Universiti Kebangsaan Malaysia, Kuala Lumpur, Malaysia; University of Glasgow, UNITED KINGDOM

## Abstract

**Introduction:**

Renin-angiotensin system inhibitors (RAS) drugs have a proteinuria-reducing effect that could prevent the progression of kidney disease in diabetic patients. Our study aimed to assess the budget impact based on healthcare payer perspective of increasing uptake of RAS drugs into the current treatment mix of standard anti-hypertensive treatments to prevent progression of kidney disease in patient’s comorbid with hypertension and diabetes.

**Methods:**

A Markov model of a Malaysian hypothetical cohort aged ≥30 years (N = 14,589,900) was used to estimate the total and per-member-per-month (PMPM) costs of RAS uptake. This involved an incidence and prevalence rate of 9.0% and 10.53% of patients with diabetes and hypertension respectively. Transition probabilities of health stages and costs were adapted from published data.

**Results:**

An increasing uptake of RAS drugs would incur a projected total treatment cost ranged from MYR 4.89 billion (PMPM of MYR 27.95) at Year 1 to MYR 16.26 billion (PMPM of MYR 92.89) at Year 5. This would represent a range of incremental costs between PMPM of MYR 0.20 at Year 1 and PMPM of MYR 1.62 at Year 5. Over the same period, the care costs showed a downward trend but drug acquisition costs were increasing. Sensitivity analyses showed the model was minimally affected by the changes in the input parameters.

**Conclusion:**

Mild impact to the overall healthcare budget has been reported with an increased utilization of RAS. The long-term positive health consequences of RAS treatment would reduce the cost of care in preventing deterioration of kidney function, thus offsetting the rising costs of purchasing RAS drugs. Optimizing and increasing use of RAS drugs would be considered an affordable and rational strategy to reduce the overall healthcare costs in Malaysia.

## Introduction

Diabetes and cardiovascular diseases are among the major chronic diseases in the Asia Pacific region and the numbers of cases are expected to grow rapidly over the coming years [1]. In this region, within a ten year time span between 1990 and 2010, the disability-adjusted-life-years of cardiovascular disease and diabetes increased by 22.6% and 69% respectively [1]. The prevalence of these diseases steadily increased from 1996 to 2015 in Malaysia with data from the National Health and Morbidity Survey reported the 2015 prevalence of diabetes at 17.5% and hypertension at 30.3% [2].

Clinically, the presence of diabetes and hypertension co-morbidity expedite the progression of kidney deterioration by seven-folds compared to an age-matched control of patients with diabetes only [3]. Naturally, increasing prevalence of end-stage renal disease (ESRD) will lead to unfavorable clinical and economic consequences. Financially, dialysis programs for ESRD consume substantial healthcare resources even in developed countries [4]; with per-patient costs of dialysis treatment in 2002 around €60,000 in European countries and US$50,000 in the United States [5, 6]. The quantum of this financial impact coupled with the increasing number of patients requiring dialysis will be devastating in developing countries with limited healthcare resources such as Malaysia. Hence, appropriate efforts to reduce or avoid this negative economic consequences should be made in Malaysia as it is heavily burdened by high dialysis rate [7]. In 2014, incidence of ESRD caused by diabetes mellitus accounted for 61% of patients with primary renal disease in Malaysia [8]. Hypertension furthermore added another 18% of new ESRD cases [8].

From the perspective of health care administrators and planners, the affordability of drugs is unarguably a major consideration in their inclusion into public reimbursement or subsidy list. Economic studies have shown promising positive evidence of cost-saving and/or cost-effectiveness of implementing early treatment of renin-angiotensin system inhibitors (RAS) drugs to prevent the progression of nephropathy in patients comorbid with diabetes and hypertension [4, 6, 7, 9–16]. Budget impact analysis additionally is a tool in estimating the expected expenditure changes in the healthcare system after adoption of the new intervention. This tool is used for budget or resources planning, forecasting and computing the impacts of introducing new treatments either as isolated assessment or used together with cost-effectiveness analyses [17]. Therefore, our study aimed to assess the budget impact based on healthcare payer perspective of increasing uptake of RAS drugs into current treatment mix of standard anti-hypertensive treatments to prevent progression of kidney disease in patients comorbid with hypertension and diabetes.

## Study design and model description

### Data source

Databases including EMBASE, PubMed and Ovid were searched from inception to June 2017 for published literature related to the effectiveness of the RAS drugs. Randomized controlled trials (RCTs) comparing the effectiveness of RAS with other antihypertensive drugs were selected. Search terms of “renin-angiotensin system inhibitors” OR “angiotensin-converting enzyme” OR “angiotensin-receptor blockers or antagonists” AND “diabetic nephropathy (ies)” OR “diabetic renal [18] disease (s)” AND “pharmacoeconomic (s)” OR “economic evaluation” OR “cost-effectiveness analysis” AND “Malaysia” OR “Asia” were utilized. The search was limited to English language articles and human studies. Publications on the economic outcomes of RAS drugs in the Malaysian situation were specifically searched.

Due to the paucity of evidence focusing on the Malaysian situation, grey literatures in publicly available databases such as government reports, policy statements, issue papers and conference proceedings were also searched.

The sources of inputted data for the model were tabulated in the S1 File.

### Model description

Our model aimed to stimulate the clinical course of a population with type 2 diabetes mellitus, hypertension and nephropathy as they progressed from microalbuminuria (MAU) to doubling serum creatinine (DSC), macro-albuminuria, onset of ESRD and death (S2 File). It was based on a Markov simulation to allow modelling of transitions between health states according to their probabilities. This allowed the description of forward disease progression, summing the costs and outcomes of all consequences. The simulation provided description of scenario between increasing uptake of RAS compared to the standard therapy. The model was designed and programmed using Microsoft Excel 2010 (Microsoft, USA) as Microsoft Excel is readily available to most researchers, especially those from countries with limited resources. Furthermore, the transparency of an Excel-based model would facilitate easy validation by other researchers.

### Patient population

Malaysia has a highly subsidized tax-based public healthcare system ensuring all citizens access to public health services with minimal fee [19]. In this model, the Malaysian cohort aged ≥30 years were used to reflect total number of adult persons eligible for the treatment (Table 1). The inclusion of cohort aged ≥30 years old would be relevant to reflect the increasing incidence and prevalence of hypertension and type 2 diabetes mellitus in this age group in Malaysia [20]. Hence, the assumptions made could be considered reasonable to describe population at risk of developing study endpoints within the time horizon of the analyses. Among this Malaysian cohort, 1,313,091 (9%) patients with both diabetes and hypertension initiated their treatment yearly; and 1,536,299 (10.53%) were on maintenance treatment.

**Table 1:** Base-case scenario for a budget impact analysis of adding drugs that inhibit renin-angiotensin system to standard antihypertensive treatments for patients with hypertension and diabetes in Malaysia.

Model parameter	Value	
*Number of persons included in the model* (*N* = 14,589,900)	*Incidence (%)*	*Prevalence (%)*
Diabetes and hypertension	9.00	10.53
MAU	0.02	4.18
Macro-albuminuria	1.65	2.41
DSC ^a^	1.65	2.41
ESRD	0.02	0.12
*Costs (Malaysian Ringgit)*		
Health states	MAU, macro-albuminuria and DSC ^a^	1,258.36
	ESRD	2,821.97
Drug acquisition	RAS	6,273.70
	Standard anti-hypertensive	4,444.35

DSC: doubling serum creatinine; ESRD: End-stage renal disease; MAU: micro-albuminuria; RAS: Renin-angiotensin system inhibitors.

^a^The incidence, prevalence and costs of treating DSC were assumed to be similar to macro-albuminuria health state.

Among existing patients, prevalence of patients with MAU was the highest at 4.18% followed by macro-albuminuria (2.41%) and doubling serum creatinine (2.41%). Data on the incidence and prevalence of DSC health state were still lacking, thus the distribution of patients in this state was assumed to be similar to those in macro-albuminuria stage. Details of values used for base-case scenario were tabulated in Table 1.

### Time horizon

Planning of economic development in Malaysia is usually prepared in the short, medium and long term. The economic planning unit is responsible to prepare macro-economic framework for a five-year time horizon, with the current being the Eleventh Malaysia Plan, spanning from 2016 to 2020 [21]. Thus, estimation of the annual cycle of budget impact for a five-year time horizon in our study would be reasonable for healthcare planning.

### Treatments

A hypothetical cohort of patients in the model received one of the following treatments;

*Standard anti-hypertensive treatment*: Standard anti-hypertensive treatment alone excluding the use of RAS drugs.
*Renin-angiotensin system inhibitors (RAS)*: Standard anti-hypertensive treatments plus RAS drugs including acetylcholine esterase inhibitors (ACEI) and angiotensin receptor blockers (ARBs).

Data for treatment mix was based on the large Malaysian survey of the national medicines utilization in 2010 [22]. This nationwide survey analyzed the utilization of prescription-only medicines in both private and public healthcare settings in Malaysia. The 2010 average percentage utilization described as defined daily dose (DDD) per 1000 population/day was used to estimate the current uptake, while the average increment of drug utilization from 2009 was used to project the new treatment uptake.

At present, the percentage of RAS utilization in public institution was estimated to be about 27.4% of the total antihypertensive utilization. The utilization of RAS was estimated to increase by 9% following the increment of its utilization from 19.89 DDD/ 1000 population/day in 2009 to 28.44 DDD/ 1000 population/day in 2010 [22].

### Transition probabilities

**Fig 1:**
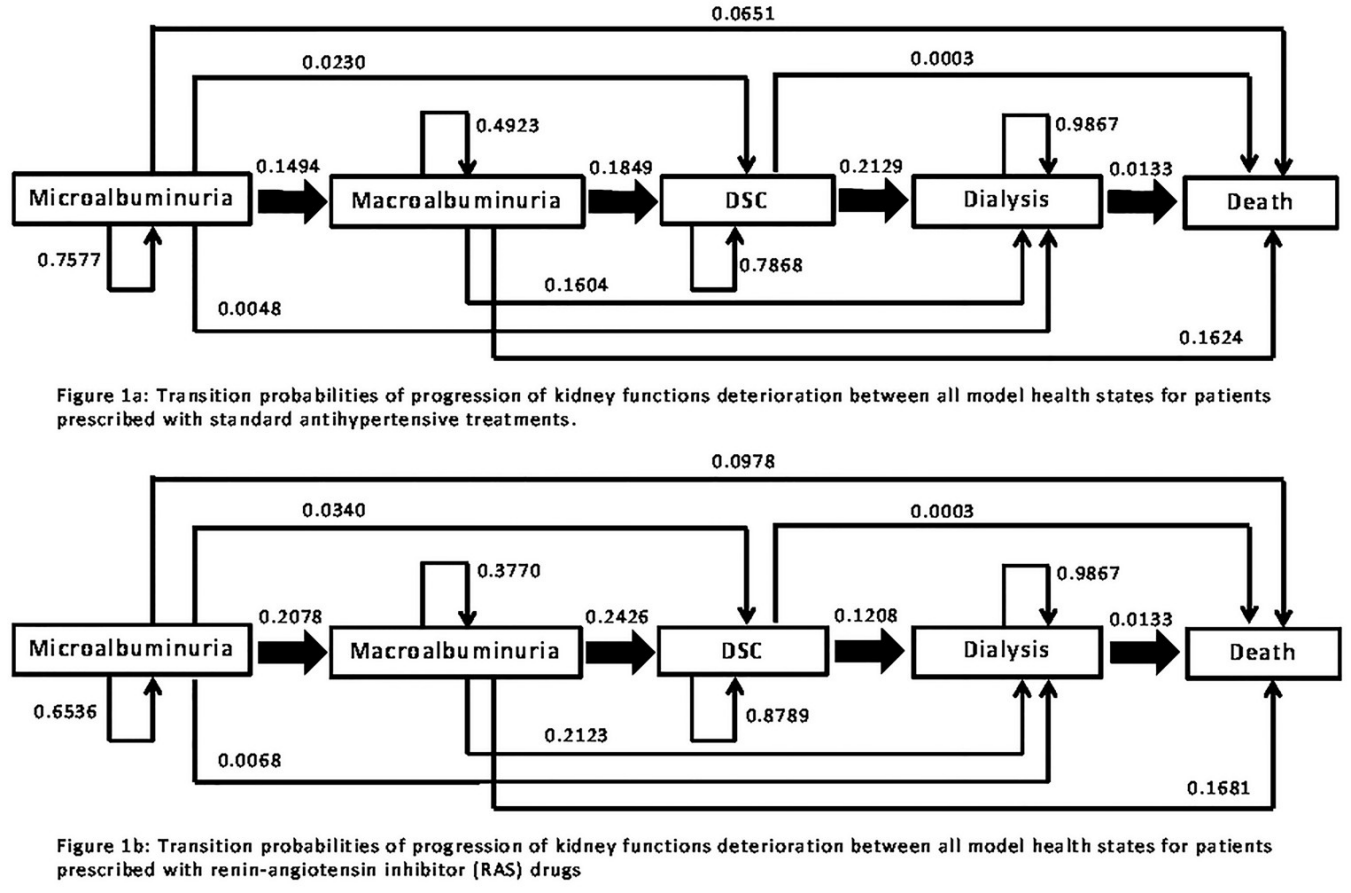
Transition probabilities of the progression of kidney function deterioration between all model health states. Fig. 1(a): For patients prescribed with standard antihypertensive treatments. Fig. 1(b): For patients prescribed with renin-angiotensin inhibitor (RAS) drugs. Microalbuminuria (MAU): 24-hour urinary albumin excretion [UAE] of 20–200 μg/minute or 30-300mg/24 hour; Macro-albuminuria: 24-hour UAE of >200 μg/minute or >300mg/24 hour; Doubling serum creatinine (DSC) from baseline; End-stage renal disease (ESRD) treated by dialysis; All-cause mortality.

Transition probabilities describe the process of kidney function deterioration from one health state to another. In our model, it followed the five stages as shown in Fig 1. The values for transition probabilities between health states were based on the probability of the timing (i.e., number of days) to transit to another health state, and were adapted from multiple large clinical trials (Fig 1) (S1 File). Furthermore, the mortality rate of ESRD was based on the report from the Malaysian National Kidney Registry, regardless of treatment strategy [8]. Mortality for DSC health state was calculated by adjusting the age- and gender-specific cause mortality values and assumed to be independent of treatment groups [23].

### Costs of treatment

As the primary aim of a budget impact analysis is to inform healthcare-decision makers, the health and costs outcomes should be specifically designed based on their perspective. Incorporation of the direct medical costs (including drug acquisition, costs of care and dialysis costs) should be considered relevant to represent the viewpoint of the health payer.

Average per-year costs of drugs were estimated from the Malaysian 2010 national annual expenditure of medicines [22]. The 2010 annual costs of RAS drugs were estimated at MYR 6,273.70 while standard anti-hypertensive was at MYR 4,444.35 (Table 1). Annual per-patient care costs for health state of MAU, macro-albuminuria and DSC were conservatively assumed to be equal, based on the direct annual costs of treating hypertension with diabetes comorbidity in an outpatient setting of urban primary medical center in Kuala Lumpur (Table 1) [24]. The cost components included the costs of administration, salary, medication, laboratory and radiology. Costs of treating ESRD were the sum of costs of treating diabetes and hypertension, and the costs for hemodialysis treatment. The costs for dialysis was based on the capital, human resources, overhead, and consumable costs of hemodialysis procedures in the Malaysia Ministry of Health hospitals [25].

Both the treatment costs and cost of care were inflated using the consumer price index (CPI) to the 2015 Malaysian Ringgit value. After inflation, the cost of hypertension and diabetes treatment was estimated at MYR 1,258.36, while ESRD treatment was at MYR 2,821.97. For international comparison, the conversion to US$ was made based on the 2015 Malaysian official exchange rate at 3.9 [26].

### Cost metrics

The aggregated cost was the sum of drug and care costs in preventing progression of kidney function loss. The incremental budget impact was calculated based on the difference between scenario of those with an increasing uptake of RAS and those on the standardized treatments. Estimates for per-member per-month (PMPM) costs were also calculated.

### Sensitivity analyses

A series of one-way sensitivity analyses were conducted to give some insight into the factors influencing the results. One-way sensitivity analyses were chosen due to its simplicity and the ability to estimate overall uncertainties of the inputted data.

Key parameters included (1) numbers of new and existing individuals in the model, (2) percentage uptake of RAS drugs, (3) costs of RAS drugs, (4) costs of ESRD, and (5) costs of DSC. Changes (±50%) of the baseline key parameters were applied to represent the worst and best case scenarios, except for parameter of percentage uptake of RAS drugs. A 50% increase in the uptake of RAS drugs was deemed desirable and possible to represent the best case scenario in the model. For example, for the costs of RAS drugs, the baseline monthly costs of MYR 522.81 was assumed to reduce to MYR 261.41 (-50% change) and increase to MYR 784.22 (+50% change) respectively for the conduct of one-way sensitivity analysis. The values for sensitivity analysis for each key parameter were tabulated in the Table 2.

**Table 2:** Key parameter values for one-way sensitivity analyses.

Key parameter	Baseline value	-50% change	+50% change
Incidence	1,313,091	656,546	1,969,637
Prevalence	1,536,316	768,158	2,304,474
Percentage uptake of RAS (%)	9.0	4.5	13.5
Costs of RAS drugs (MYR)	522.81	261.41	784.22
Costs of ESRD	2822.00	1411.00	4233.00
Costs of DSC	1258.00	629.00	1887.00

MYR: Malaysian Ringgit; RAS: Renin-angiotensin system inhibitors; ESRD: End-stage renal disease; DSC: doubling serum creatinine

## Results

### Budget impact

**Fig 2:**
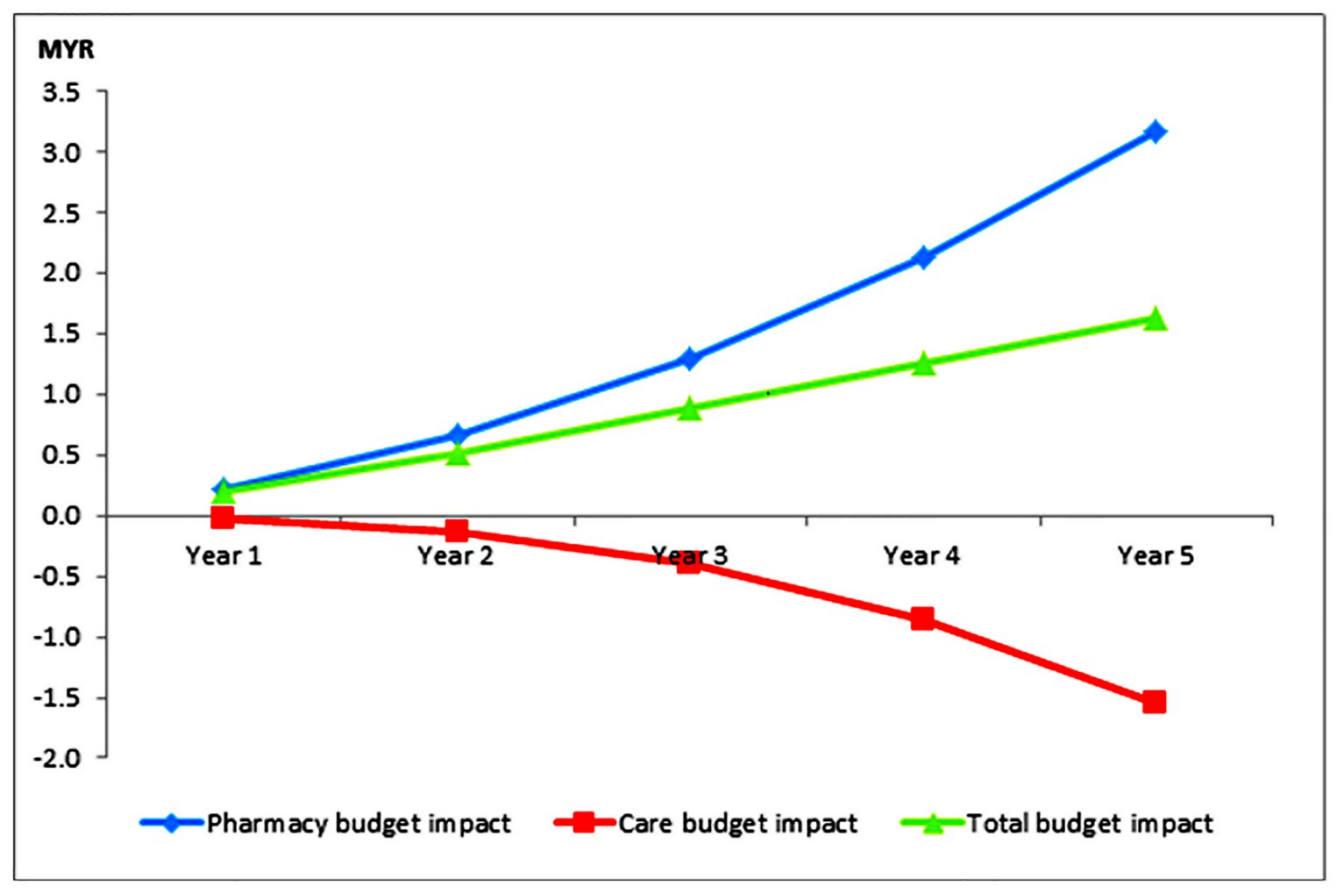
Incremental per-member per-month (MYR) cost impact after increasing uptake of renin-angiotensin system inhibitor drugs.

The baseline costs of managing progression of kidney disease in patients with diabetes and hypertension was MYR 2.57 billion. After an annual 9% uptake of RAS drugs, the total costs of treatment was projected to increase to MYR 4.89 billion (PMPM of MYR 27.95) at Year 1 and MYR 16.26 billion (PMPM of MYR 92.89) at Year 5. These represented increments of the total budget ranged from MYR 34.18 million at Year 1 to MYR 284.18 million at Year 5, compared to the budget before RAS uptake (Table 3). There was a trend of reduction in the care costs annually while drug costs showed an upward trend (Fig 2). The increment in the total costs of managing kidney disease was mainly due to the costs of drug acquisition (ranging from MYR 39.09 million at Year 1 to MYR 555.79 million at Year 5). Reduction in care costs (ranging from MYR 4.91 million to MYR 271.61 million) would further offset the rising costs of drug acquisition.

**Table 3:** Budget impact analysis of increasing uptake of renin-angiotensin system inhibitors (RAS) drugs to standard anti-hypertensive treatments for patient with hypertension and diabetes in Malaysia.

Budget impact	Year 1	Year 2	Year 3	Year 4	Year 5
Treatment costs (‘000,000)	39.09	114.22	225.38	372.57	555.79
Costs of care (‘000,000)	4.91	24.39	70.14	141.35	271.61
Total (‘000,000)	34.18	89.83	155.24	221.22	284.18
Per-member-per-month (PMPM)	0.20	0.51	0.89	1.26	1.62

The negative sign (-) means costs saving as compared to the current uptake of treatment.

Using per-member-per-month (PMPM) metrics, the total budget impact ranged from MYR 0.20 at Year 1 to MYR 1.62 at Year 5. This represented an incremental range from 0.70% (at Year 1) to 1.78% (at Year 5) as compared to budget prior the uptake of RAS in the health plan.

The budget for drug acquisition will increase by an average of 10% (range: 3.33–16.65%) after increasing RAS uptake in the health plan. In contrast, the care budget will reduce by an average of 1.02% (with a range of 0.13–2.15%).

### Sensitivity analyses

**Fig 3:**
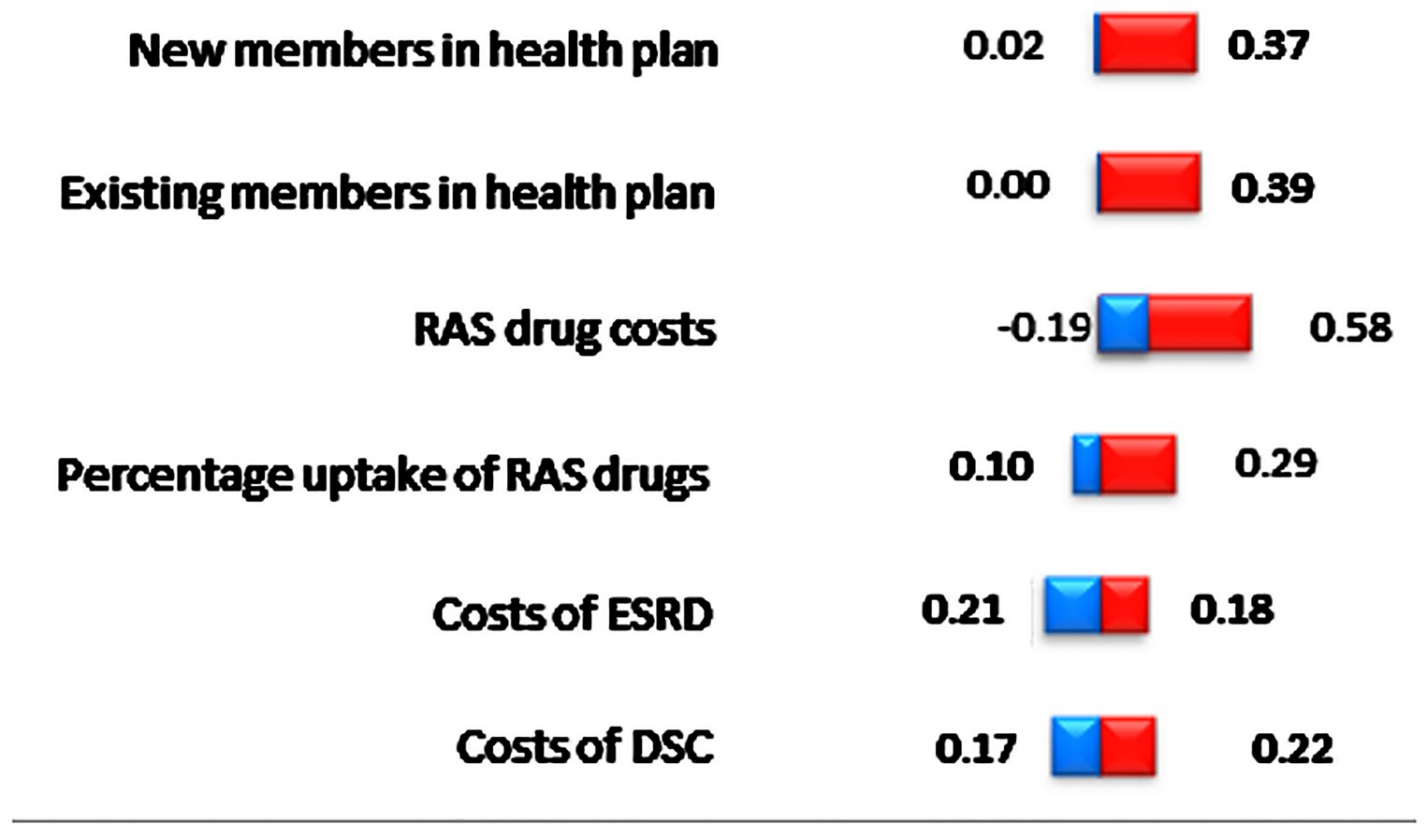
Tornado diagram of incremental per-member per-month costs (MYR) after increasing uptake of renin-angiotensin system inhibitor drugs.

Summary of one-way sensitivity analyses was presented in Table 4 and the Tornado diagram in Fig 3. The sensitivity analyses across six sets of key parameters ranges from PMPM of MYR 0.003 to 0.578. This indicated the budget impact range from MYR -32.85 million (PMPM of MYR -0.19) to MYR 101.22 million (PMPM of MYR 0.58), compared to baseline at MYR 34.18 million (PMPM MYR 0.20).

**Table 4:** Sensitivity analyses: Incremental total and per-member per-month (PMPM) costs (MYR) after one-year of increasing uptake of RAS drugs.

Key parameter	-50% change	+50% change
Incidence	-3.29 (0.019)	65.08 (0.372)
Prevalence	-0.49 (0.003)	67.87 (0.388)
Percentage uptake of RAS drugs	-17.09 (0.098)	51.27 (0.293)
Costs of RAS drugs (MYR)	-32.85 (-0.188)	101.22 (0.578)
Costs of ESRD	36.06 (0.206)	32.31 (0.185)
Costs of DSC	30.34 (0.173)	38.02 (0.217)

MYR: Malaysian Ringgit; RAS: Renin-angiotensin system inhibitors; ESRD: End-stage renal disease; DSC: Doubling serum creatinine

Total costs is reported in million (MYR); PMPM costs is reported in a bracket; The baseline incremental total costs at year 1 is reported at MYR 34.18 million.

RAS costs had the greatest impact to the incremental PMPM costs of all parameters tested for the sensitivity analyses,. Cost saving of MYR 0.19 (PMPM costs) was reported with a 50% reduction in the RAS costs. This would represent a reduction of the total budget costs from baseline of MYR 34.18 million to achieve a cost saving of MYR 32.85 million at Year 1. In contrast, doubling the RAS cost from MYR 522.81 would increase the total budget to MYR 101.22 million (PMPM of MYR 0.58).

Analyzed independently, the incremental cost of care was very sensitive to the changes in the key parameters of DSC costs. If the DSC cost was reduced by 50%, it would reduce the care costs from MYR 4.91 million to MYR 8.75 million. If the DSC cost was doubled, it would still save MYR 1.08 million at Year 1. Furthermore, the care budget impact would have parallel adjustment with the ±50% changes in the key parameters of treatment uptake and prevalence. If the numbers of existing patients were halved from the base-case, it would save MYR 2.46 million at Year 1, representing 50% reduction from care budget at baseline. On the other hand, the cost-saving of care budget would increase by 50% (i.e., MYR 7.37 million) when the prevalence were doubled.

Consecutively, drug budget was very sensitive to the ±50% changes in the RAS costs. If the RAS cost was reduced by 50%, there would be cost saving of MYR 27.94 million in the drug budget at Year 1. However, the drug budget would increase from the baseline at MYR 39.09 million to MYR 106.13 million if the RAS cost was doubled.

## Discussion

The use of RAS in patients comorbid with diabetes and hypertension had shown to be effective in reducing proteinuria (the earliest clinical evidence of nephropathy), and cardiovascular events in many clinical trials [4, 6, 7, 9–16]. Consequently, most hypertension guidelines have recommended the use of RAS as first line therapy for diabetic patients. Additionally, studies had shown the use of RAS to be cost-effective and/or cost-saving in preventing the progression of kidney function deterioration in patients with diabetes and hypertension, especially if started at the early stage of kidney disease [4, 6, 7, 9–16]. Considering these positive clinical and economic impacts, the RAS uptake is possibly still low and may need to be optimized in Malaysia. This was shown indirectly from an audit of diabetes management in Asian patients treated by specialists. The audit reported that the incidence of neuropathy was still high (~33%) in diabetic patients in Asia with a significant proportion of patients had unsatisfactory control of blood pressure and glycemic level [27].

An important reason for lower than expected uptake of RAS drugs in developing countries would be the higher costs of these drugs. For example, in 2010, the utilization of 13.71 DDD/1000 population/day for perindopril consumed MYR 83.89 million of healthcare resources as compared to MYR 8.22 million for 26.74 DDD/1000 population/day of amlodipine [22]. Similar to our study, the acquisition costs of optimizing RAS was projected to increase up to 16.65% (at Year 5) as compared to scenario before the uptake. The increase in the drug budget was similar to that reported in a UK study [28]. This study reported that the expenditure for purchasing ARBs after its introduction in the UK accounted for 9.3% of the total of 213% increment in the anti-hypertensive drugs expenditure (over a 10 year period). In our opinion, this is still considered small considering the RAS positive outcomes in preventing kidney function deterioration, and subsequent reduced in the overall costs of ESRD treatment.

In healthcare planning, drug acquisition should not be the only type of cost or aspect to determine the affordability of treatment. Our analyses showed that although the drug acquisition cost of purchasing RAS would further increase the budget, the effectiveness of RAS in delaying the kidney function deterioration eventually would reduce the overall clinical and financial burdens of the patients. The projected care costs would continuously reduce following RAS uptake, with large reduction seen at year 4 of their utilization. This latent reduction of care expenditure is supported by the results of another economic modelling evaluation reporting cost-saving and/or cost-effectiveness of RAS could only be achieved after 3.5 years of RAS utilization [6]. Hence, our outcomes were able to show that increasing uptake of RAS would only have mild impact on the total health budget.

Like any other studies, there are limitations in our analyses due to the assumptions made in the model. Thus, our analyses should be interpreted with appropriate caution. Currently, there are no comprehensive trials to predict the impact of diabetes and hypertension in the progression of nephropathy in Malaysia. Therefore, the first limitation of the present study was the use of evidence from published literature to evaluate the clinical outcomes of anti-hypertensive drugs in type 2 diabetes mellitus. Adoption of these data from multinational clinical trials would provide the required evidence that is reasonably acceptable especially in countries with limited availability of data. However, these trials lack information on the effect of controlling blood glucose level to prevent nephropathy. Tight glycemic control has been shown to reduce or reverse the risk of MAU, and reduce the risk of macro-albuminuria, crucial to prevent the progression of diabetic nephropathy [29]. Therefore, further evaluation of the cumulative effects of tight glycemic control and the use of RAS in preventing progression of nephropathy is indispensable.

In our study, we also assumed treatment costs of patient in the health state of MAU, macro-albuminuria and DSC were considered to be equal. However, in real-life situation, the treatment cost of patient in the later stages of kidney function deterioration is possibly higher due to additional monitoring and consultation visits. For example, the use of RAS might increase the risk of hyperkalemia requiring additional monitoring especially in patients at their later stage of kidney disease. Furthermore, the cost might be underestimated as we did not include the cost of hospitalization in the model. We further relied on the local cost of hemodialysis conducted 12 years ago, although adjustments were made to represent the current costs of treatment. We are aware that these limitations could be a potential threat to generalization of the findings. However, the approach can be still considered relevant due to very limited availability of the published local costs data.

Finally, due to a lack of a suitable cohort and other technical constraints, we could not perform a validation of our model. However, the similar results obtained from other studies would provide some indirect evidence of the validity of our model. The similar time period to achieve cost saving between our current study and a published study [6] can in some way act as a surrogate validation of our model. Furthermore, sensitivity analyses were conducted to address uncertainties and assumptions made in our current model.

Overall, although our study suffers from several limitations, the estimated budget impact obtained in our study would support and promote wider use of RAS drugs in patients with hypertension, diabetes and nephropathy. After increasing the budget between 0.70–1.78% in the initial five years, increased use of RAS would subsequently provide positive long-term health and financial benefits to the patients and healthcare system. Therefore, optimizing use of RAS drugs should be considered a rational strategy to reduce the prevalence of ESRD and its heavy financial burden. Furthermore, although our analysis is specifically designed based on the Malaysian cohort, considering the similar trend of increasing prevalence of hypertension and diabetes, and the effectiveness of RAS globally, the trends of our budget outcomes could be used as supportive evidence for healthcare planning in other countries, especially of those with similar tax-based payments systems and developing countries.

## Conclusion

This budget impact model showed that optimizing RAS utilization would have a small impact to the overall healthcare budget. However, the long-term positive health consequences of RAS treatment will further reduce the cost of care in preventing deterioration of kidney function offsetting the initial rising costs of purchasing RAS drugs. Optimization of RAS drugs would therefore be considered an affordable and rational strategy to reduce the overall healthcare costs in the long term, and provide cost-effective treatment to the population.

## Supporting information

S1 File Input data and data sources for a budget impact analysis of adding drugs that inhibits renin-angiotensin system (RAS) to standard antihypertensive treatments in patients with diabetes, and hypertension. (PDF)

S2 File Summary of modelling data and results for study evaluating the budget impact of increasing prescription of renin-angiotensin system inhibitors drugs to standard anti-hypertensive treatments in patients with diabetes and hypertension in a hypothetical cohort of Malaysian population. (PDF)
