# Supplementary material for: Budget impact analysis of increasing prescription of renin-angiotensin system inhibitors drugs to standard anti-hypertensive treatments in patients with diabetes and hypertension in a hypothetical cohort of Malaysian population
Source: PLoS One. 2019 Feb 28;14(2):e0212832. doi: 10.1371/journal.pone.0212832 (PMC6394912; doi:10.1371/journal.pone.0212832)
Supplement: S1 File — (PDF) [file pone.0212832.s001.pdf]

Budget impact analysis of increasing prescription of renin-angiotensin system inhibitors drugs to standard anti-hypertensive treatments in patients with diabetes and hypertension in a hypothetical cohort of Malaysian population

PLOS ONE

Nurul-Ain Mohd-Tahir, PhD[1, 2], Shu-Chuen Li, PhD[1]

Corresponding Author: Shu-Chuen Li, School of Biomedical Sciences and Pharmacy, University of Newcastle, University Drive, Callaghan, NSW 2308, Australia

E-mail: Shuchuen.Li@newcastle.edu.au

Electronic Supplementary materials

S1 File: Input data and data sources for a budget impact analysis of adding drugs that inhibits renin-angiotensin system (RAS) to standard antihypertensive treatments in patients with diabetes, and hypertension.

S1 Table: Input data and data sources for a budget impact analysis of adding drugs that inhibits renin-angiotensin system (RAS) to standard antihypertensive treatments in patients with diabetes, and hypertension.

**Input data**	**Data source**
Total number of persons aged ≥18 years in the population of the health plan	Department of Statistics, Malaysia
Numbers of new and existing members in the model	Wong, 2005; Annemans et al., 2008; Mohamed & Mafauzy, 2008; Fesiul & Azmi, 2013
Distribution of individuals, by health state	Kong et al., 2006; Wong, 2005; Mohamed & Mafauzy, 2008; Goh & Ong, 2015
Transition probabilities between health states of diseases progression	Palmer et al., 2003; Rodby et al., 2003; Palmer et al., 2005; Strippoli et al., 2006; Annemans et al., 2008; Wu et al., 2013
Age- and sex-specific all-cause mortality	WHO, 2015
Cost of anti-hypertensive drugs	PSD, 2014
Annual per patient care costs	Hooi et al., 2005; Azimatun Noor et al., 2014
