# Supplementary material for: Budget impact analysis of increasing prescription of renin-angiotensin system inhibitors drugs to standard anti-hypertensive treatments in patients with diabetes and hypertension in a hypothetical cohort of Malaysian population
Source: PLoS One. 2019 Feb 28;14(2):e0212832. doi: 10.1371/journal.pone.0212832 (PMC6394912; doi:10.1371/journal.pone.0212832)
Supplement: S2 File — (PDF) [file pone.0212832.s002.pdf]

Budget impact analysis of increasing prescription of renin-angiotensin system inhibitors drugs to standard anti-hypertensive treatments in patients with diabetes and hypertension in a hypothetical cohort of Malaysian population

PLOS ONE

Nurul-Ain Mohd-Tahir, PhD[1, 2], Shu-Chuen Li, PhD[1]

Corresponding Author: Shu-Chuen Li, School of Biomedical Sciences and Pharmacy, University of Newcastle, University Drive, Callaghan, NSW 2308, Australia

E-mail: Shuchuen.Li@newcastle.edu.au

Electronic Supplementary materials

S2 FILE: Summary of modelling data and results for study evaluating the budget impact of increasing prescription of renin-angiotensin system inhibitors drugs to standard anti-hypertensive treatments in patients with diabetes and hypertension in a hypothetical cohort of Malaysian population

Model Overview

Budget-Impact Analysis

This model investigates the potential budget and health impact of including a renin-angiotensin system inhibitor (RAS) drugs on the formulary of a Malaysian Ministry of Health (MOH) payer that currently includes standard anti-hypertensives control drugs (Standard anti-HPT) for prevention of progression of kidney disease in patient with hypertension and diabetes comorbidities. Budget and health outcomes over a 5-year period are reported annually based on a hypothetical mix of standard anti-hypertensive or RAS drugs treatment.

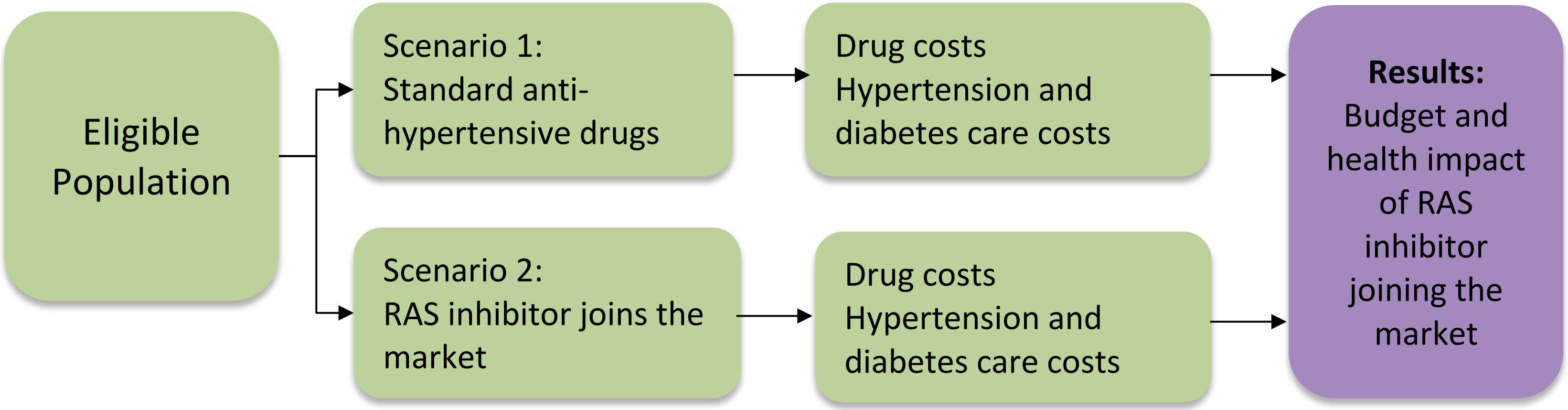

Markov Model Structure

The model uses a Markov framework with a 1-year cycle time. Patients may transition between five health states: microalbuminuria, macroalbuminuria, doubling serum creatinine, dialysis and death. Patients have a chance of progressing to death from any health state.

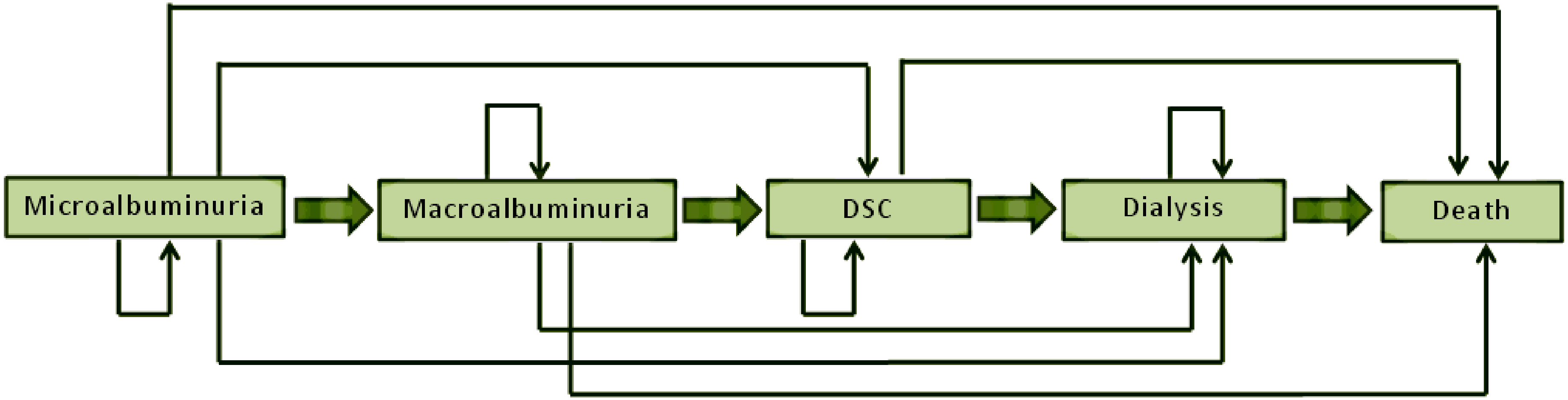

Population

Patients Living with Condition

To arrive at the size of the modeled population, this table begins with the population in the overall health plan. Then, filters are applied to estimate the size of the currently treated population and the number of people initiating treatment each year.

Parameter	User Input				Number of People
Total number of persons in the population of the health plan	14,589,900				14,589,900
Percent of diabetes and hypertension individuals receiving anti-hypertensive maintenance treatment within health plan	10.53%	x	14,589,900	=	1,536,316
Percent of diabetes and hypertension individuals in the health plan who begin anti-hypertensive treatment each year	9.00%	x	14,589,900	=	1,313,091

	Existing Patients		New Patients	
	Default	User Input	Default	User Input
Distribution of individuals by health state				
MAU	4.18%	45.29%	0.02%	0.57%
Macroalbuminuria	2.41%	26.13%	1.65%	49.17%
**DSC	2.41%	26.13%	1.65%	49.17%
Dialysis (ESRD)	0.12%	1.25%	0.02%	0.70%
Death	0.11%	1.20%	0.01%	0.40%
	9.23%	100.00%	3.36%	100.00%

Assumption: The model assumes all diabetic patients are eligible for Standard anti-HPT, and RAS.

Treatment Mix

Uptake of RAS Over Time

This table displays the uptake of RAS drugs to prevent kidney disease progression for the next 5 years.

Year	Default	User Input
Year 1	9.00%	36.40%
Year 2	18.0%	45.40%
Year 3	27.0%	54.40%
Year 4	36.0%	63.40%
Year 5	45.0%	72.40%

Source: based on average 9% increment of RAS drugs utilization from 2009 to 2010 (Malaysian Statistics of Medicine, 2010)

Treatment Mix

The budget-impact model compares two scenarios: (1) a scenario in which only standard anti-hypertensive treatments are available, and (2) a scenario in which RAS treatment also becomes available. This table displays the treatment mix for each scenario in the current year and for the first 5 years after RAS becomes available. In Scenario 2, RAS uptake is assumed to affect the usage of standard anti-hypertensive treatments equally.

Comparator	Current Year	Year 1	Year 2	Year 3	Year 4	Year 5
**Scenario 1: Standard anti-hypertensive treatments and RAS in the market**						
Standard anti-hypertensive	72.60%	72.60%	72.60%	72.60%	72.60%	72.60%
RAS	27.40%	27.40%	27.40%	27.40%	27.40%	27.40%
Total	100.0%	100.0%	100.0%	100.0%	100.0%	100.0%
**Scenario 3: increasing uptake of RAS drugs**						
Standard anti-hypertensive	72.60%	63.60%	54.60%	45.60%	36.60%	27.60%
RAS	27.40%	36.40%	45.40%	54.40%	63.40%	72.40%
Total	100.0%	100.0%	100.0%	100.0%	100.0%	100.0%

Costs

Costs

This table provides monthly HPT treatment costs and annual other HPT care costs.

Parameters	Default	User Input
**Annual drug costs**		
Standard anti-hypertensive	$370.36	$4,444.35
RAS drugs	$522.81	$6,273.70
**Annual per-patient care costs, by health state**		
MAU	$1,258.36	$1,258.36
macroalbuminuria	$1,258.36	$1,258.36
DSC	$1,258.36	$1,258.36
ESRD	$2,821.97	$2,821.97

Efficacy

Annual Transitions Between Health States

The table below displays the annual (year 1) probabilities of transitioning between the model health states.

Transition	Std anti-HPT User Input	RAS User Input
microalbuminuria to macroalbuminuria	0.1494	0.2078
microalbuminuria to DSC	0.0230	0.0340
microalbumniuria to ESRD	0.0048	0.0068
macroalbuminuria to DSC	0.1849	0.2426
macroalbumniuria to ESRD	0.1604	0.2123
DSC to ESRD	0.2129	0.1208
**Transitions to death state**		
Mortality from microalbuminuria	0.0651	0.0978
Mortality from macroalbuminuria	0.1624	0.1681
Mortality from DSC	0.0003	0.0003
Mortality from ESRD	0.0133	0.0133

Summary of Transition Probabilities

The table below summarizes the annual probabilities of transitioning between all model health states.

Standard anti-hypertensive treatments: Transition Probabilities

		To				
		MAU	macroalbuminuria	DSC	ESRD	Death
From	MAU	0.7577	0.1494	0.0230	0.0048	0.0651
	macroalbumniuria	0.0000	0.4923	0.1849	0.1604	0.1624
	DSC	0.0000	0.0000	0.7868	0.2129	0.0003
	ESRD	0.0000	0.0000	0.0000	0.9867	0.0133
	Death	0.0000	0.0000	0.0000	0.0000	1.0000

RAS drugs: Transition Probabilities

		To				
		MAU	macro albuminuria	DSC	ESRD	Death
From	MAU	0.6536	0.2078	0.0340	0.0068	0.0978
	macroalbumniuria	0.0000	0.3770	0.2426	0.2123	0.1681
	DSC	0.0000	0.0000	0.8789	0.1208	0.0003
	ESRD	0.0000	0.0000	0.0000	0.9867	0.0133
	Death	0.0000	0.0000	0.0000	0.0000	1.0000

Markov Calculations

Standard anti-hypertensive alone

Time (Years)	Existing Patients					New Patients				
	MAU	Macro albuminuria	DSC	ESRD	Death	MAU	Macro albuminuria	DSC	ESRD	Death
0	0.4529	0.2613	0.2613	0.0125	0.0120	0.0057	0.4917	0.4917	0.0070	0.0040
1	0.3432	0.1963	0.2643	0.1121	0.0842	0.0043	0.2429	0.4779	0.1904	0.0844
2	0.2600	0.1479	0.2521	0.2000	0.1400	0.0033	0.1202	0.4210	0.3286	0.1269
3	0.1970	0.1117	0.2317	0.2759	0.1837	0.0025	0.0597	0.3535	0.4332	0.1511
4	0.1493	0.0844	0.2075	0.3405	0.2184	0.0019	0.0298	0.2892	0.5123	0.1668
5	0.1131	0.0639	0.1823	0.3944	0.2464	0.0014	0.0149	0.2331	0.5718	0.1787

Markov Calculations

Increasing uptake of RAS)

Time (Years)	Existing Patients: MAU	Existing Patients: Macro-albuminuria	Existing Patients: DSC	Existing Patients: ESRD	Existing Patients: Death	New Patients: MAU	New Patients: Macro-albuminuria	New Patients: DSC	New Patients: ESRD	New Patients: Death
0	0.4529	0.2613	0.2613	0.0125	0.0120	0.0057	0.4917	0.4917	0.0070	0.0040
1	0.2960	0.1926	0.3084	0.1025	0.1005	0.0037	0.1866	0.5516	0.1707	0.0874
2	0.1935	0.1341	0.3278	0.1813	0.1633	0.0024	0.0711	0.5302	0.2747	0.1216
3	0.1265	0.0908	0.3272	0.2482	0.2073	0.0016	0.0273	0.4833	0.3502	0.1376
4	0.0827	0.0605	0.3139	0.3046	0.2383	0.0010	0.0106	0.4314	0.4097	0.1472
5	0.0540	0.0400	0.2934	0.3519	0.2607	0.0007	0.0042	0.3818	0.4586	0.1547

Budget-Impact Analysis Calculations

Summary of Parameters and Therapy-specific Budget-impact Analysis

Summary of Treatment Mixes

Scenario 1: Standard anti-hypertensive and RAS

Time (Years)	Standard anti-hypertensive	RAS
0	72.60%	27.40%
1	72.60%	27.40%
2	72.60%	27.40%
3	72.60%	27.40%
4	72.60%	27.40%
5	72.60%	27.40%

Scenario 2: increasing uptake of RAS drugs

Time (Years)	Standard anti-hypertensive	RAS
0	72.60%	27.40%
1	63.60%	36.40%
2	54.60%	45.40%
3	45.60%	54.40%
4	36.60%	63.40%
5	27.60%	72.40%

Summary of Therapy-specific Budget-Impact Analysis

	MAU		Macroalbuminuria		DSC		ESRD		Death	
	Std anti-HPT	RAS	Std anti-HPT	RAS	Std anti-HPT	RAS	Std anti-HPT	RAS	Std anti-HPT	RAS
0	695,850	695,850	401,386	401,386	401,386	401,386	19,226	19,226	18,468	18,468
1	534,763	462,325	947,175	941,533	1,051,633	1,119,415	181,292	166,548	134,544	159,586
2	412,708	309,694	1,191,801	1,096,642	1,660,440	1,873,570	566,412	511,735	331,137	370,859
3	320,226	209,933	1,293,995	1,123,401	2,181,856	2,568,815	1,114,677	975,323	564,834	598,116
4	250,153	144,730	1,330,488	1,112,759	2,608,861	3,182,947	1,782,608	1,521,735	816,570	826,509
5	197,059	102,113	1,337,985	1,095,198	2,949,952	3,717,891	2,538,081	2,132,363	1,078,695	1,054,205

	Drug costs		Care Costs	
	Std anti-HPT	RAS	Std anti-HPT	RAS
0	$568,994,043	$803,198,993	$1,940,061,211	$1,940,061,211
1	$1,055,313,738	$1,489,693,859	$3,699,746,535	$3,645,180,530
2	$1,541,633,433	$2,176,188,725	$5,706,879,406	$5,571,401,267
3	$2,027,953,128	$2,862,683,591	$7,922,418,210	$7,662,642,855
4	$2,514,272,823	$3,549,178,458	$10,302,368,064	$9,881,957,611
5	$3,000,592,518	$4,235,673,324	$12,806,128,595	$12,202,559,986

Budget-Impact Analysis Calculations: Scenario 1 - Standard anti-HPT and RAS

Distribution of Patients Over Time

Time (Years)	MAU	Microalbuminuria	DSC	ESRD	Death
0	695,502	401,185	401,185	19,216	18,459
1	514,915	945,629	1,070,205	177,252	141,405
2	384,482	1,165,727	1,718,838	551,431	342,021
3	290,006	1,247,252	2,287,883	1,076,494	573,954
4	221,267	1,270,830	2,766,160	1,711,129	819,294
5	171,044	1,271,461	3,160,367	2,426,915	1,071,985

Cost and Health Outcomes

	Drug costs			Distribution of All Patients (%)					
Time (Years)	Std anti-HPT	RAS	Care Costs	MAU	Micro-albuminuria	DSC	ESRD	Alive	Number Alive (N)
0	$413,089,675	$219,674,925	$1,940,061,211	45.3	26.1	26.1	1.3	98.8	1,517,089
1	$766,157,774	$408,176,117	$3,684,795,449	18.1	33.2	37.6	6.2	95.0	2,708,002
2	$1,119,225,872	$596,275,711	$5,669,758,396	9.2	28.0	41.3	13.2	91.8	3,820,477
3	$1,472,293,971	$784,375,304	$7,851,239,763	5.3	22.8	41.8	19.7	89.5	4,901,636
4	$1,825,362,070	$972,474,897	$10,187,175,600	3.3	18.7	40.7	25.2	87.9	5,969,387
5	$2,178,430,168	$1,160,574,491	$12,640,750,796	2.1	15.7	39.0	30.0	86.8	7,029,787

Budget-Impact Analysis Calculations: Scenario 2 - increasing uptake of RAS drugs

Distribution of Patients Over Time

Time (Years)	MAU	Macroalbuminuria	DSC	ESRD	Death
0	695,850	401,386	401,386	19,226	18,468
1	508,396	945,121	1,076,305	175,926	143,659
2	365,939	1,148,599	1,757,201	541,589	349,171
3	260,227	1,201,192	2,392,362	1,038,869	582,940
4	183,315	1,192,448	2,972,831	1,617,214	822,872
5	128,318	1,162,207	3,505,940	2,244,342	1,060,964

Cost and Health Outcomes

Time (Years)	Drug costs: Std anti-HPT	Drug costs: RAS	Care Costs	Distribution of All Patients (%): MAU	Macro-albuminuria	DSC	ESRD	Alive	Number Alive (N)
0	$413,089,675	$219,674,925	$1,940,061,211	45.3	26.1	26.1	1.3	98.8	1,517,848
1	$671,179,537	$542,248,565	$3,679,884,509	17.8	33.2	37.8	6.2	95.0	2,705,748
2	$841,731,854	$987,989,681	$5,645,372,331	8.8	27.6	42.2	13.0	91.6	3,813,328
3	$924,746,626	$1,557,299,874	$7,781,100,417	4.8	21.9	43.7	19.0	89.4	4,892,650
4	$920,223,853	$2,250,179,142	$10,035,827,837	2.7	17.6	43.8	23.8	87.9	5,965,809
5	$828,163,535	$3,066,627,486	$12,369,144,922	1.6	14.3	43.3	27.7	86.9	7,040,807

Budget-Impact Analysis Results

Budget-Impact Analysis Results

Outcome	Current Year	Year 1	Year 2	Year 3	Year 4	Year 5
**Annual Cost Outcomes**						
Scenario 1: Standard anti-HPT and RAS drugs						
Drug costs: Standard anti-HPT	$413,089,675	$766,157,774	$1,119,225,872	$1,472,293,971	$1,825,362,070	$2,178,430,168
Drug costs: RAS	$219,674,925	$408,176,117	$596,275,711	$784,375,304	$972,474,897	$1,160,574,491
Total drug costs	$632,764,600	$1,174,333,891	$1,715,501,583	$2,256,669,275	$2,797,836,967	$3,339,004,659
Care costs	$1,940,061,211	$3,684,795,449	$5,669,758,396	$7,851,239,763	$10,187,175,600	$12,640,750,796
Total costs	$2,572,825,811	$4,859,129,341	$7,385,259,979	$10,107,909,038	$12,985,012,567	$15,979,755,455
Scenario 2: increasing uptake of RAS drugs						
Drug costs: Standard anti-HPT	$413,089,675	$671,179,537	$841,731,854	$924,746,626	$920,223,853	$828,163,535
Drug costs: RAS	$219,674,925	$542,248,565	$987,989,681	$1,557,299,874	$2,250,179,142	$3,066,627,486
Total drug costs	$632,764,600	$1,213,428,102	$1,829,721,536	$2,482,046,500	$3,170,402,995	$3,894,791,021
Care costs	$1,940,061,211	$3,679,884,509	$5,645,372,331	$7,781,100,417	$10,035,827,837	$12,369,144,922
Total costs	$2,572,825,811	$4,893,312,611	$7,475,093,867	$10,263,146,917	$13,206,230,832	$16,263,935,943

Outcome	Current Year	Year 1	Year 2	Year 3	Year 4	Year 5
**Budget impact**						
Pharmacy budget impact	$0	$39,094,211	$114,219,953	$225,377,225	$372,566,028	$555,786,362
Care budget impact	$0	-$4,910,940	-$24,386,065	-$70,139,346	-$151,347,763	-$271,605,874
Total budget impact	$0	$34,183,271	$89,833,888	$155,237,879	$221,218,265	$284,180,488

Outcome	Current Year	Year 1	Year 2	Year 3	Year 4	Year 5
Health Outcomes						
Scenario 1: Standard anti-HPT and RAS						
Number Alive	1,517,089	2,708,002	3,820,477	4,901,636	5,969,387	7,029,787
% with MAU	45.29%	18.07%	9.24%	5.30%	3.26%	2.11%
% with Macroalbuminuria	26.13%	33.19%	28.01%	22.78%	18.72%	15.69%
% with DSC	26.13%	37.56%	41.29%	41.29%	40.75%	39.01%
% with ESRD	1.25%	6.22%	13.25%	19.66%	25.21%	29.96%
Scenario 2: increasing uptake of RAS						
Number Alive	1,517,848	2,705,748	3,813,328	4,892,650	5,965,809	7,040,807
% with MAU	45.29%	17.84%	8.79%	4.75%	2.70%	1.58%
% with Macroalbuminuria	26.13%	33.17%	27.59%	21.94%	17.57%	14.35%
% with DSC	26.13%	37.77%	42.22%	43.69%	43.79%	43.27%
% with ESRD	1.25%	6.17%	13.01%	18.97%	23.82%	27.70%

All outcomes presented are undiscounted, in alignment with the recommendations for budget-impact analysis.

Per-Member-Per-Month Results

Outcome	Current Year	Year 1	Year 2	Year 3	Year 4	Year 5
**Cost Outcomes**						
Scenario 1: Standard anti-HPT, and RAS						
Drug costs: Standard anti-HPT	$2.36	$4.38	$6.39	$8.41	$10.43	$12.44
Drug costs: RAS	$1.25	$2.33	$3.41	$4.48	$5.55	$6.63
Total drug costs	$3.61	$6.71	$9.80	$12.89	$15.98	$19.07
Care costs	$11.08	$21.05	$32.38	$44.84	$58.19	$72.20
Total costs	$14.70	$27.75	$42.18	$57.73	$74.17	$91.27
Scenario 2: increasing uptake of RAS						
Drug costs: Standard anti-HPT	$2.36	$3.83	$4.81	$5.28	$5.26	$4.73
Drug costs: RAS	$1.25	$3.10	$5.64	$8.89	$12.85	$17.52
Total drug costs	$3.61	$6.93	$10.45	$14.18	$18.11	$22.25
Care costs	$11.08	$21.02	$32.24	$44.44	$57.32	$70.65
Total costs	$14.70	$27.95	$42.70	$58.62	$75.43	$92.89
Budget impact						
Pharmacy budget impact	$0.00	$0.22	$0.65	$1.29	$2.13	$3.17
Care budget impact	$0.00	-$0.03	-$0.14	-$0.40	-$0.86	-$1.55
Total budget impact	$0.00	$0.20	$0.51	$0.89	$1.26	$1.62

Sensitivity analyses calculation

Sensitivity analyses calculation

	Key parameters values	Budget impact budget			
		Pharmacy	Care	Total	Per-member per-month
Prevalence		$39,094,211	-$4,910,940	$34,183,271	$0.1952
Base case	1,536,316	$39,094,188	-$4,910,938	$34,183,249	$0.1952
-50%	768,158	$2,950,245	-$2,455,469	$494,775	$0.0028
+50%	2,304,474	$75,238,132	-$7,366,408	$67,871,724	$0.3877
Incidence					
Base case	1,313,091	$39,094,211	-$4,910,940	$34,183,271	$0.1952
-50%	656,545	$39,094,211	-$4,910,940	$34,183,271	$0.1952
+50%	1,969,636	$39,094,211	-$4,910,940	$34,183,271	$0.1952
RAS costs					
Base case	$522.81	$39,094,648	-$4,910,940	-$32,852,734	-$0.1876
-50%	$261.41	-$27,941,794	-$4,910,940	$34,183,271	$0.1952
+50%	$784.22	$106,131,090	-$4,910,940	$101,220,150	$0.5781

Treatment uptake					
Base case	4.50%	$39,094,211	-$4,910,940	$34,183,271	$0.1952
-50%	9.00%	$19,547,105	-$2,455,470	$17,091,635	$0.0976
+50%	13.50%	$58,641,316	-$7,366,410	$51,274,905	$0.2929
Costs of DSC					
Base case	$1258	$39,094,211	-$4,913,136	$34,181,074	$0.1952
-50%	$629	$39,094,211	-$8,750,274	$30,343,936	$0.1733
+50%	$1887	$39,094,211	-$1,075,998	$38,018,212	$0.2171
Costs of ESRD					
Base case	$2822	$39,094,211	-$4,913,136	$34,181,074	$0.1952
-50%	$1411	$39,094,210	-$3,038,637	$36,055,573	$0.2059
+50%	$4233	$39,094,210	-$6,783,323	$32,310,887	$0.1846
